# Changes in the Gut Microbiota of Urban Subjects during an Immersion in the Traditional Diet and Lifestyle of a Rainforest Village

**DOI:** 10.1128/mSphere.00193-18

**Published:** 2018-08-29

**Authors:** Kelly V. Ruggles, Jincheng Wang, Angelina Volkova, Monica Contreras, Oscar Noya-Alarcon, Orlana Lander, Hortensia Caballero, Maria G. Dominguez-Bello

**Affiliations:** a Department of Medicine, New York University Langone Medical Center, New York, New York, USA; b Department of Biochemistry and Microbiology, Rutgers University, New Brunswick, New Jersey, USA; c Center for Biophysics and Biochemistry, Venezuelan Institute of Scientific Research (IVIC), Caracas, Venezuela; d Instituto de Medicina Tropical, Universidad Central de Venezuela, Caracas, Venezuela; e Amazonic Center for Research and Control of Tropical Diseases (CAICET), Puerto Ayacucho, Venezuela; f Department of Anthropology, Venezuelan Institute of Scientific Research (IVIC), Caracas, Venezuela; g Department of Anthropology, New York University, New York, New York, USA; h Department of Anthropology, Rutgers University, New Brunswick, New Jersey, USA; University of Wisconsin-Madison

**Keywords:** Amerindian, diet, microbiome

## Abstract

People living traditional lifestyles have higher gut microbiota diversity than urban subjects. We hypothesized that shifting lifestyles from an urban environment to a traditional rainforest village would lead to changes in the microbiota of visitors, which would become more similar to the microbiota of villagers. Here, we characterized at different time points the microbiota of 7 urban visitors (5 adults and 2 children) staying in a rainforest Amerindian village for 16 days and compared them with a reference collection of samples from age-matched local villagers. We performed a 16S rRNA gene survey of samples from multiple body sites (including fecal, oral, nasal, and skin samples) using Illumina MiSeq sequencing. The main factor segregating the microbiotas of each body site was the human group (i.e., visitors versus villagers), with the visitor microbiota tending to have lower alpha diversity; the lowered alpha diversity was statistically significant in the microbiota of skin and in the children’s fecal and oral microbiota. During the rainforest period, all visitors experienced microbiota changes within their personal cloud of variation. For all body sites, the microbiota conformations in the visitor children better matched the microbiota conformations in villagers of the same age than did those of the visitor adults, which showed a lower “microbiota age” than the microbiota of the villagers. The results suggest higher stability in the adult microbiota, with the less resilient children’s microbiota responding more to dietary changes.

**IMPORTANCE** Despite the limitations of a small study, our results evidence higher resilience of the gut microbiota with respect to dietary manipulation in adults than in children and urge further studies to understand the extent of microbiota plasticity in response to dietary changes and the mechanisms underlying microbiota resilience. These studies are relevant to the potential of future human pre- and probiotics in preventing or curing microbiota-associated diseases.

## Abstract (precis)

Despite the limitations of a small study, our results evidence higher resilience of the gut microbiota with respect to dietary manipulation in adults than in children and urge further studies to understand the extent of microbiota plasticity in response to dietary changes and the mechanisms underlying microbiota resilience. These studies are relevant to the potential of future human pre- and probiotics in preventing or curing microbiota-associated diseases.

## INTRODUCTION

The microbiota influences our physiological responses and disease risks (1). Different microbiota conformations present early in life are associated with different phenotypes such as increased body weight (2, 3). The transfer of phenotypes with the microbiota in mice has provided a strong proof of causation of various diseases such as obesity (4–7), glucose intolerance (5), metabolic syndrome (8), type 1 diabetes (9), and colitis (10).

Diet is an important modulator of the gut microbiota (5, 7, 11, 12). Dietary molecules and products of digestion that bypass the small intestine reach the colon and serve as substrates for microbes, which produce bioactive compounds with effects both on the microbial ecosystem and on the host. For example, plant cell walls, which are resilient with respect to mammalian enzymes, select for fiber degrader bacteria that were shown to ferment plant polymers into short-chain fatty acids that could nourish colonocytes and other host tissues (13). Both low-plant-fiber diets (14) and high-fat diets (15) have been shown to reduce gut microbiota diversity over several generations. Reintroducing dietary plant fibers did not restore the higher diversity (14). In addition, other lifestyle factors, such as circadian rhythms (16) and individual hygiene practice (17, 18), have been shown to influence the commensal microbiota in human and experimental animals.

People in rural or remote villages with a traditional lifestyle have higher microbiota diversity (19–22). Their traditional diet is more abundant with dietary fiber and less-processed food, which contains high levels of plant cell wall carbohydrates and low levels of soluble sugars and fat (14, 20). Since rapid alterations in the human gut microbiome after diet changes have been reported (23), we hypothesized that immersion in a setting with a traditional diet and lifestyle (such as life sharing, circadian rhythms, and hygiene practices) would reshape the microbiota of urban subjects, making it more diverse and similar to that of local villagers. Here we determined the structure of the microbiota in urban subjects at multiple body sites, during a 16-day stay in a remote village in the rainforest south of Venezuela.

## RESULTS

Samples from a total of 7 urban subjects (5 adults and 2 children) were collected from different body sites (i.e., nasal cavity, mouth, and skin) and from feces during a 16-day visit to a rainforest village in the state of Bolivar in Venezuela, close to the Brazilian border. The urban visitors consumed only a traditional low-fat/high-fiber unprocessed diet, adopted the circadian activities of the locals (for example, going to bed early since there was no electricity, waking up early with the sunrise, and getting 8 to 9 h of sleep), and bathed in the rivers without using soaps or shampoo. Samples from age-matched local villagers (11 adults and 27 children) were also collected at one time point. Microbial DNA was extracted, and a survey of 16S rRNA gene data was performed on a total of 327 samples (Table S1).

10.1128/mSphere.00193-18.9 TABLE S1  Numbers of samples from visitors and villagers from each body site and at each time point. Download TABLE S1, DOCX file, 0.01 MB. Copyright © 2018 Ruggles et al. 2018 Ruggles et al. This content is distributed under the terms of the Creative Commons Attribution 4.0 International license.

**FIG 1:**
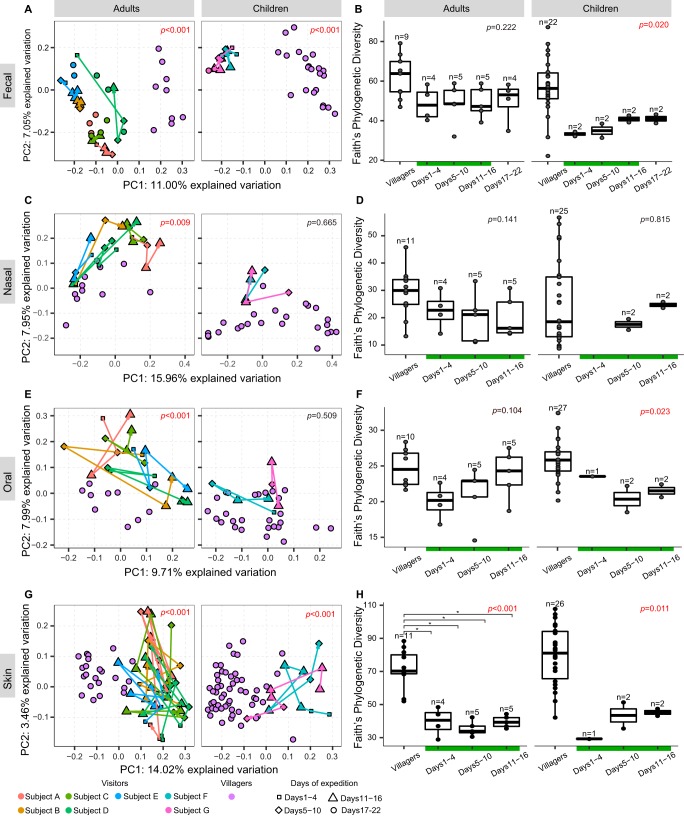
Comparison of microbial communities in adult and children visitors and villagers. (A, C, E, and G) Principal-coordinate analysis based on unweighted UniFrac index of microbial communities, split by time periods and subjects. Arrowed lines connect samples from the same individual across time, showing the individual trajectories of microbiota changes based on beta diversity during the rainforest visit, from day 1 to day 16. The visitors returned to the city of Caracas on day 17. ANOSIM test were performed to compare visitors and villagers; the overall significance is indicated by the *P* value (in red where *P* < 0.05). (B, D, F, and H) Box plots of Faith’s phylogenetic diversity index comparing visitors to villagers at different time periods. Kruskal-Wallis tests with *post hoc* Dunn’s test using Benjamini-Hochberg correction were performed to determine significance. *P* values indicate overall significance determined using the Kruskal-Wallis test; asterisks (*) and brackets indicate groups shown to be significantly different by Dunn’s test with adjusted *P* values.

The results show that with respect to body sites, the skin and fecal microbiotas had the highest alpha diversity, followed by the nasal and oral microbiotas (Fig. 1). Overall, large variations were seen within all body sites and time points. The main factors segregating the microbiotas of each body site were the human groups (i.e., visitors versus villagers; Fig. 1). In addition, principal coordinate 1 (PC1) from the principal-coordinate analysis (PCoA) (Fig. 1; see also Fig. S1 in the supplemental material) segregated fecal and skin microbiotas, and PC2 segregated nasal and oral sites. Microbiota alpha diversity tended to be lower in urban visitors than in villagers, but the difference was statistically significant only in the microbiota of skin and children’s fecal and oral microbiotas (Fig. 1). Visitor children showed a trend toward increasing alpha diversity during the rainforest period (Fig. 1; see also Fig. S2), although the data were not statistically significant.

10.1128/mSphere.00193-18.1 FIG S1  Principal-coordinate analysis based on Bray-Curtis distances of microbial communities, split by time points and subjects. Arrowed lines connect samples collected from the same individual while the individual was living in Kanarakuni village during day 1 to day 16. The visitors returned to the city of Caracas on day 17. ANOSIM tests were performed to compare data from visitors and villagers; the overall significance is indicated by the *P* value. Download FIG S1, PDF file, 0.2 MB. Copyright © 2018 Ruggles et al. 2018 Ruggles et al. This content is distributed under the terms of the Creative Commons Attribution 4.0 International license.

10.1128/mSphere.00193-18.2 FIG S2  Faith’s phylogenetic diversities for microbial communities in different body sites across time. The visitors stayed at Kanarakuni village from day 1 to day 16. Download FIG S2, PDF file, 0.1 MB. Copyright © 2018 Ruggles et al. 2018 Ruggles et al. This content is distributed under the terms of the Creative Commons Attribution 4.0 International license.

During the rainforest stay, there was no evidence of divergence of individual microbiotas from the baseline (samples noted with squares in Fig. 1A, C, E, and G) or of interindividual convergence with respect either to the conformation of the villagers (Fig. S3) or to reducing interindividual distances among visitors, with the exception of the skin microbiota, as was evidenced by the smaller interindividual skin microbiota distances seen at the end of the stay (Fig. S4).

10.1128/mSphere.00193-18.3 FIG S3  Box plot of unweighted UniFrac distance data corresponding to local villagers calculated by comparisons with themselves and with visitors at different time points. Kruskal-Wallis tests with *post hoc* Dunn’s test using Benjamini-Hochberg correction were performed to determine significance. *P* values indicate overall significance determined using the Kruskal-Wallis test; asterisks (*) and brackets indicate groups shown to be significantly different by Dunn’s test with adjusted *P* values. Download FIG S3, PDF file, 0.2 MB. Copyright © 2018 Ruggles et al. 2018 Ruggles et al. This content is distributed under the terms of the Creative Commons Attribution 4.0 International license.

10.1128/mSphere.00193-18.4 FIG S4  Box plot of interindividual unweighted UniFrac distances among visitors at different time points. Kruskal-Wallis tests with *post hoc* Dunn’s test using Benjamini-Hochberg correction were performed to determine significance. *P* values indicate overall significance determined using the Kruskal-Wallis test; asterisks (*) and brackets indicate groups shown to be significantly different by Dunn’s test with adjusted *P* values. Download FIG S4, PDF file, 0.2 MB. Copyright © 2018 Ruggles et al. 2018 Ruggles et al. This content is distributed under the terms of the Creative Commons Attribution 4.0 International license.

The fecal beta diversity changed over time within visitors’ individual variation clouds in the PCoA plot (Fig. 1A), reflecting the strong individuality of the intestinal microbiotas. In children, there was a gain in alpha diversity during the rainforest visit. All visitors reported a change in fecal color from brown to clear yellow, within the first week of arrival in the rainforest, consistent with the lower levels of bile secretion associated with low-fat diets. Results of linear discriminant analysis effect size (LEfSe) analyses of human group-discriminant taxa showed that the adult visitors had lower fecal levels of *Treponema*, *Succinivibrio*, and *Ruminobacter* spp. and higher fecal levels of *Bacteroides*, *Blautia*, *Faecalibacterium*, *Coprococcus*, *Roseburia*, unknown genera from the *Rikenellaceae* and S24-7 families, and *Clostridiales* spp. than the adult villagers (Fig. S5A). The two visiting children had lower proportions of fecal *Escherichia* and of unknown *Ruminococcaceae* and *Bacteroidales* genera than the local children and higher proportions of *Bacteroides*, *Faecalibacterium*, *Blautia*, *Clostridium*, *Coprococcus*, *Ruminococcus*, *Lachnospira*, *Bifidobacterium*, and unknown *Lachnospiraceae* and *Rikenellaceae* genera (Fig. S5B). Levels of *Faecalibacterium* significantly increased in the visiting children during the rainforest stay (Kruskal-Wallis test with Dunn’s *post hoc* multiple-comparison text, corrected using the Benjamini-Hochberg procedure).

10.1128/mSphere.00193-18.5 FIG S5  Heat map of the group-discriminant taxa on the genus level with a linear discriminant analysis (LDA) score of >2 based on LEfSe analysis of microbial communities in villagers and visitors. The visitors were in Kanarakuni village during day 1 to day 16 (indicated by the green bar) and returned to the city of Caracas on day 17. Download FIG S5, PDF file, 0.2 MB. Copyright © 2018 Ruggles et al. 2018 Ruggles et al. This content is distributed under the terms of the Creative Commons Attribution 4.0 International license.

Significant visitor-villager differences were detected for the nasal and oral microbiota beta diversities in adults but not in children (Fig. 1C and E). There were several taxa with different levels of abundance between visitors and villagers, including the visiting children having lower nasal *Streptococcus* and *Haemophilus* levels and higher *Alloococcus* and *Corynebacterium* levels (Fig. S5C) and the visiting adults having higher oral *Haemophilus* levels (Fig. S5D).

The skin microbiotas of adult visitors had significantly lower diversity than those of villagers (Fig. 1H), with a lower representation of *Kocuria* spp. and an unknown Streptophyta sp. and higher levels of *Acinetobacter*, *Enhydrobacter*, and an unknown Stramenopiles sp. (Fig. S5E). Visiting children had higher levels of skin *Acinetobacter*, an unknown Stramenopiles sp., and *Enhydrobacter* than local children (Fig. S5F). During the rainforest stay, skin Stramenopiles proportions decreased to a statistically significant extent in adult visitors (at day 11 to day 16 in relation to days 1 to 4; Kruskal-Wallis test with Dunn’s *post hoc* multiple-comparison test, corrected using the Benjamini-Hochberg procedure).

**FIG 2:**
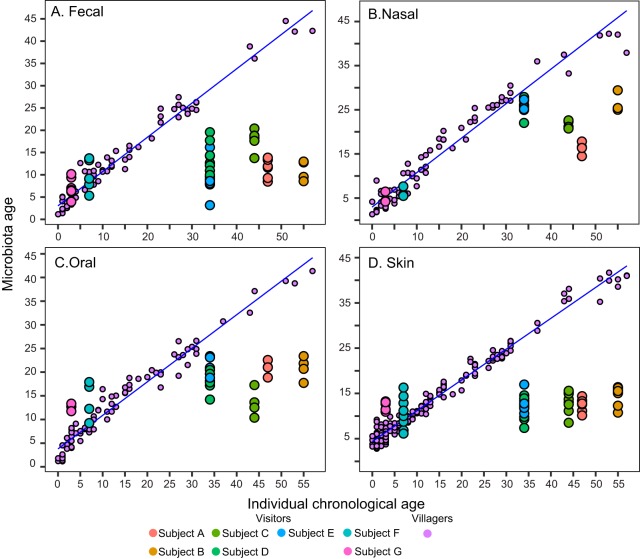
Predicted microbiota age against the actual age of visitors and villagers. (A) Fecal samples. (B) Nasal samples. (C) Oral samples. (D) Skin samples. The prediction was made by constructing a random forest model using the villagers as the training set. The blue line in each panel illustrates a simple linear regression curve using samples from the training set. Data points for visitors are from multiple dates.

Finally, with the purpose of estimating how much deviation the visitors’ microbiotas had in relation to the microbiota structure for chronological age, we used the local villagers’ microbiotas to train a random forest model, which we call “microbiota age,” similarly to the maturity analysis in Bangladeshi children that was previously reported (24). The random forest models fitted quite well for fecal and nasal microbiotas (pseudo-*R*^2^ = 55.23% and 49.96%, respectively) and fairly well for oral and skin microbiotas (pseudo-*R*^2^ = 36.16% and 27.07%, respectively). The analysis showed that for all body sites, visitor adults—but not children—showed a lower predicted “microbiota age” than was shown by the villagers (Fig. 2). This apparent microbiota “immaturity” in adult visitors did not improve during the rainforest visit (Fig. S6).

10.1128/mSphere.00193-18.6 FIG S6  Predicted microbiota age among visitors in different body sites across time. The visitors stayed at Kanarakuni village from day 1 to day 16. Download FIG S6, PDF file, 0.1 MB. Copyright © 2018 Ruggles et al. 2018 Ruggles et al. This content is distributed under the terms of the Creative Commons Attribution 4.0 International license.

## DISCUSSION

The results of this study show relatively limited changes in the gut microbiota (taxa structure) of urban subjects—particularly adults—during an immersion in the traditional diet and lifestyle of a rainforest village. Although diet has been shown to drive convergence of the gut microbiome (gene structure) (25), there seem to be limits to the plasticity of the microbiota imposed by the existing taxon diversity, which tended to be lower in the visitors than in the villagers. The changes in the microbiota of adult visitors occurred mostly within individual variation clouds, and only the skin microbiota—and not the gut, oral, or nasal microbiota—converged among adult visitors, suggesting a lack of taxon transmission or acquisition of new diversity; therefore, the microbiota plasticity was modeled by diet, within the existing individual diversity. Nevertheless, microbiota redundancy should cause diet sharing to result in functional convergence of the microbiome. Gut microbiota plasticity was more evident in the visitor children, who increased their fecal alpha diversity during their visit. It was interesting that this occurred despite the age of the children −4 to 6 years old—which was beyond that at which the child fecal microbiota has been shown to come to resemble the adult fetal microbiota (19). Additional research is needed to sort out the effect of individual factors (e.g., diet, behavior, day-night cycles, clothes, chlorine presence, and physiology) on microbiota composition.

The fecal color change to light yellow reported by the visitors likely corresponded to low levels of bile acid secretion, consistent with low-fat-diet intake. High levels of plant cell wall carbohydrates (cassava and fruits) and low levels of soluble sugars (fruits and some honey) in the diet likely increased the production of short-chain fatty acids (26, 27), but those factors were not measured in this study. Turnbaugh et al. demonstrated in mice that switching from a low-fat, plant polysaccharide-rich diet to a high-fat, high-sugar “Western” diet shifted the structure of the microbiota within a single day (7). In this human study, we observed much higher resilience in the adult fecal microbiotas than in those of the children. This finding is in line with a previous report that shifting to a plant-based diet (such as the change experienced by the visitors going to the rainforest in this experiment) did not cause significant changes in microbiotas compared to the strong increase seen in shifting to an animal-based diet, suggesting that the directionality of the dietary change could affect the degree of microbiota shifting (23). But a bigger study, controlling also for the baseline diet, is needed to better characterize the effect of diet changes. Finally, in comparing visitors to villagers, the presence of intestinal protozoa and helminths in villagers (Table S2) is likely to have an effect on the composition of their fecal microbiota (28).

10.1128/mSphere.00193-18.10 TABLE S2  Detection of parasites among villagers; 4 of 11 adults and 10 of 27 children were examined. Download TABLE S2, DOCX file, 0.01 MB. Copyright © 2018 Ruggles et al. 2018 Ruggles et al. This content is distributed under the terms of the Creative Commons Attribution 4.0 International license.

The observed mismatch between the microbiotas and the chronological ages of the visitors, in relation to villagers, in which adults appeared to have “immature” microbiotas, is consistent with their alpha diversity being lower than that of the villagers. Age increases the diversity of the microbiota and functional genes (19, 29), and an increase of resistance and resilience with age is evidenced by the resistance to transfer of phenotypes via the microbiota as recipients grow older (3, 10, 30–32). The “microbiota age” analysis generalizing the regression model from one population to another needs to be framed in the context of two human groups with different microbiotas. If the microbiotas of visitors are sufficiently different, the model will yield low-confidence predictions for those subjects. The results show that the comparison of the microbiota of adult visitors to that of age-matched locals renders a low predicted microbiota age for the chronological age. Interestingly, this apparent “immaturity” did not occur in the two visitor children.

Limitations of this study included the low subject number among the visitors, especially the visiting children, the lack of time series data in the reference group of age-matched villagers, and the lack of a control with respect to the baseline diet, which would account for some individual variability. Although the study was limited in size, the results highlight the importance of more studies to improve our understanding of the age windows of microbiota plasticity. More studies of dietary effects on the human microbiota are needed in children, since they do show a capacity to gain alpha diversity, suggesting higher microbiota plasticity. Humans are exposed to more chemically diverse and more homogeneous diets and lifestyles (high sharing) under rainforest conditions than under urban conditions (with social stratification), and these low variability settings offer opportunities for additional controlled studies. Future studies should use metabolomics and metagenomics to identify effectors (interacting microbes and phytochemicals) influencing the microbiota and should use conventionalized germfree mice to study the consequent phenotypes associated with different microbiotas or derived strains.

## MATERIALS AND METHODS

### Subjects and samples.

A total of 7 urban subjects (5 adults aged 34 to 55 and 2 children aged 3 and 7) visited a rainforest village in the upper Caura River region in the state of Bolivar at the Venezuelan border with Brazil in October 2015, under Venezuelan Institute of Scientific Research (IVIC) institutional review board (IRB) approval (project Dir0229/10 approval granted to M. Contreras). The visit consisted of a 16-day immersion in the local Amerindian lifestyle, including their diet. The diet consisted of daily intake of cassava (Yucca), fish, and a diversity of fruits, with a small portion (about two bites) of game meat three times per week. Morning, noon, and dinner meals were similar. The visitors did not use soaps or toothpaste or have access to chlorinated water but bathed in the river, as the locals did. Prior to and after the rainforest visit, the visitors stayed at Caracas, the capital city of Venezuela. Age-matched local villagers (11 adults aged 30 to 60 years and 27 children aged 2 to 8 years) were recruited as the reference populations. Samples were taken from nose, mouth, skin (on the right arm and right hand), and feces by swabbing and were immediately frozen in dry shippers in the field. Local villagers were sampled once, and visitors were sampled multiple times (i.e., 5 to 8 times) across the duration of the study (see Fig. S7 in the supplemental material).

10.1128/mSphere.00193-18.7 FIG S7  Diet study dates. Download FIG S7, PDF file, 0.2 MB. Copyright © 2018 Ruggles et al. 2018 Ruggles et al. This content is distributed under the terms of the Creative Commons Attribution 4.0 International license.

### 16S rRNA gene survey.

Microbial DNA was extracted from collected samples using a Qiagen DNeasy PowerSoil HTP 96 kit and amplified using primers targeting the V4 region of the 16S rRNA genes according to the modified protocol described in the Earth Microbiome Project website (http://press.igsb.anl.gov/earthmicrobiome/protocols-and-standards/). Prepared amplicon libraries were sequenced at the Genome Technology Center of the New York University School of Medicine using Illumina MiSeq sequencing instruments and paired-end 150 chemistry.

### Data analysis.

Analyses of the 16S rRNA sequencing data were performed using the QIIME pipeline (v1.9) (33) and the R package phyloseq (34). Paired-end reads from Illumina MiSeq were merged and quality trimmed (minimum quality score of 20), and the operational taxonomic units (OTUs) were picked using an open-reference strategy with a threshold of 97% identity to the Greengenes database (v13_8). For comparisons across samples with different sequencing depths, analysis of fecal communities was rarefied to 10,000 reads per sample, analysis of oral and nasal communities was rarefied to 4,000 reads per sample, and analysis of skin communities was rarefied to 5,000 reads per sample. These depths were deemed appropriate on the basis of the rarefication curve shown in Fig. S8.

10.1128/mSphere.00193-18.8 FIG S8  Rarefaction analysis of Faith’s phylogenetic diversity (PD). Download FIG S8, PDF file, 0.1 MB. Copyright © 2018 Ruggles et al. 2018 Ruggles et al. This content is distributed under the terms of the Creative Commons Attribution 4.0 International license.

The unweighted/weighted UniFrac distances (35) and the Bray-Curtis similarity data were calculated to obtain the pairwise beta diversity, which was further evaluated by the analysis of similarity (ANOSIM) test (36) using the R package vegan (37) to test the significance with 999 permutations. The alpha diversity analysis was performed using Faith’s phylogenetic diversity (38), Shannon index data, and observed OTUs. To determine significant differences in diversity, the Kruskal-Wallis test with the *post hoc* Dunn’s test using Benjamini-Hochberg correction was performed. Linear discriminant analysis effect size (LEfSe) was used to detect overrepresented bacterial taxa in comparisons (linear discriminant analysis [LDA] score of >2.0) (39).

The microbiota age of individuals was estimated by constructing a random forest model using local villagers as the reference (24, 40). A preliminary random forest regression model was built by using the age of villagers as the response variable and the scaled abundance of all taxa in the villagers as the predictive variable. The number of trees to grow was set to 10,000, and the number of taxa randomly sampled at each split was set to 1/3 of the total number of taxa. This preliminary model was rebuilt 100 times, and data corresponding to the importance of all taxa were averaged across the 100 runs. A feature selection step was then performed to determine the number of taxa to include in the final model, which was determined based on 5-fold cross-validation; the number of taxa that generated the lowest cross-validation error was selected. The final random forest regression model was built on a subset of predictive variables which were selected based on their importance and the number of taxa to be included. The random forest modeling natively incorporated cross-validation into the model building process, and the pseudo-*R*-squared value, calculated from the cross-validation errors, can be used to evaluate the goodness of the model fit.

The description of statistical results in this article was constructed by providing an *a priori* significance level, which was compared to the *P* value of a specific test. Unless otherwise noted, a value of 0.05 was used to determine the statistical significance. The specific statistical tests that were conducted and the *P* values are presented in the figures to avoid repetitiveness in the main text.

### Accession number(s).

The original sequences with relevant metadata can be retrieved from the public database Qiita (study identifier [ID] 11874) or through the European Nucleotide Archive (accession number ERP110254).
