# Supplementary material for: Changes in the Gut Microbiota of Urban Subjects during an Immersion in the Traditional Diet and Lifestyle of a Rainforest Village
Source: mSphere. 2018 Aug 29;3(4):e00193-18. doi: 10.1128/mSphere.00193-18 (PMC6115531; doi:10.1128/mSphere.00193-18)
Supplement: TABLE S1 [file sph004182633st1.docx]

Table S1 Number of samples from each body site and time point, in visitors and villagers.

| **Body Site** | **Villagers** | **Visitors** | | | |
| --- | --- | --- | --- | --- | --- |
|  |  | **Days1-4** | **Days5-10** | **Days11-16** | **Days17-22** |
| *Adult* |  |  |  |  |  |
| Feces | 9 | 4 | 6 | 11 | 11 |
| Nose | 11 | 5 | 7 | 9 | 0 |
| Mouth | 10 | 5 | 7 | 10 | 0 |
| Skin | 10 | 10 | 14 | 20 | 0 |
| *Children* |  |  |  |  |  |
| Feces | 22 | 2 | 2 | 5 | 5 |
| Nose | 25 | 0 | 2 | 4 | 0 |
| Mouth | 27 | 1 | 2 | 4 | 0 |
| Skin | 53 | 2 | 4 | 8 | 0 |
| **Total Villagers:** | **167** | **Total Visitors:** | | **160** | |
