# Supplementary figures and images for: Changes in the Gut Microbiota of Urban Subjects during an Immersion in the Traditional Diet and Lifestyle of a Rainforest Village
Source: mSphere. 2018 Aug 29;3(4):e00193-18. doi: 10.1128/mSphere.00193-18 (PMC6115531; doi:10.1128/mSphere.00193-18)

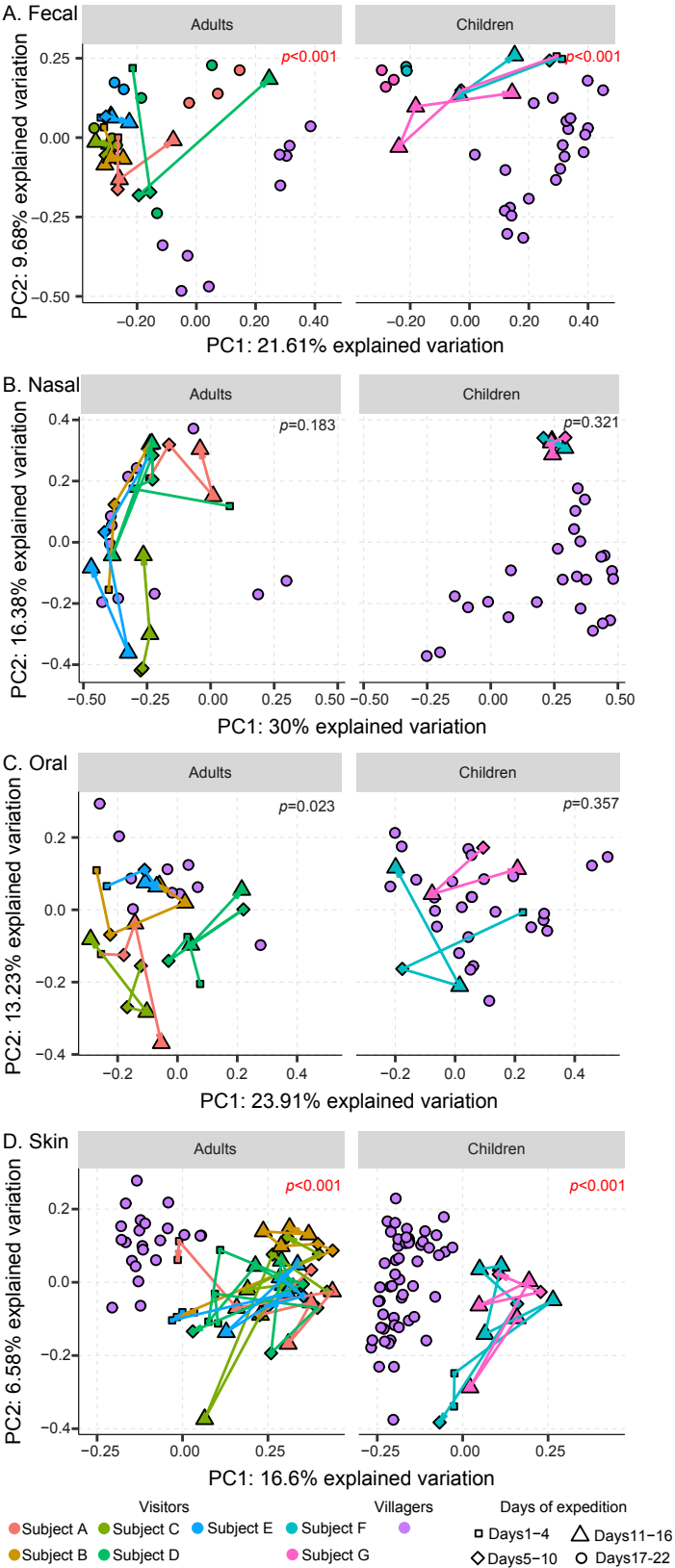

Supplement: FIG S1 [file sph004182633sf1.pdf]

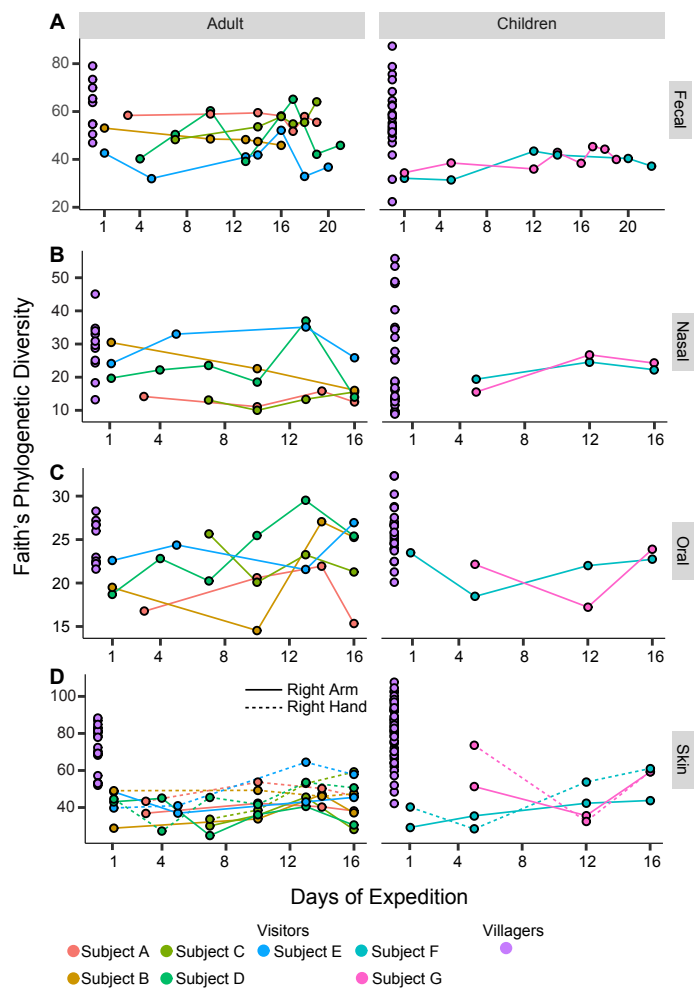

Supplement: FIG S2 [file sph004182633sf2.pdf]

## A. Fecal

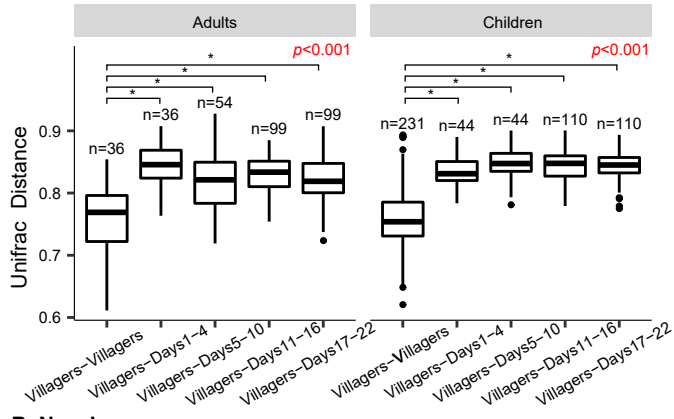

## B. Nasal

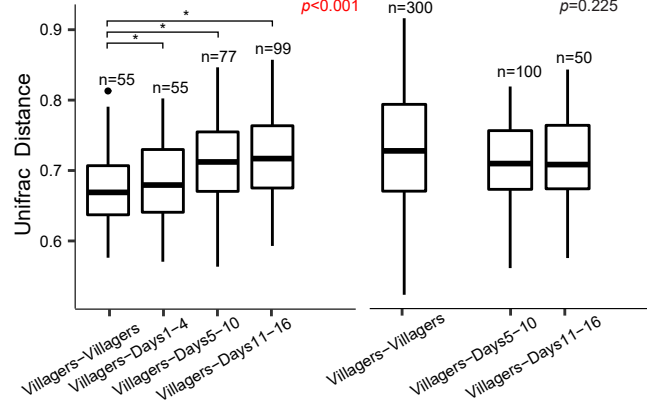

## C. Oral

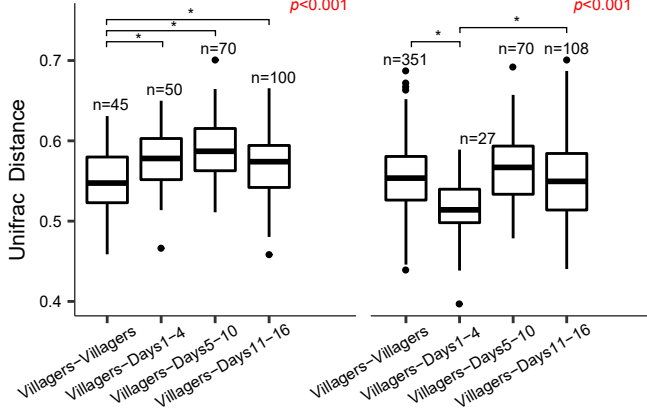

## D. Skin

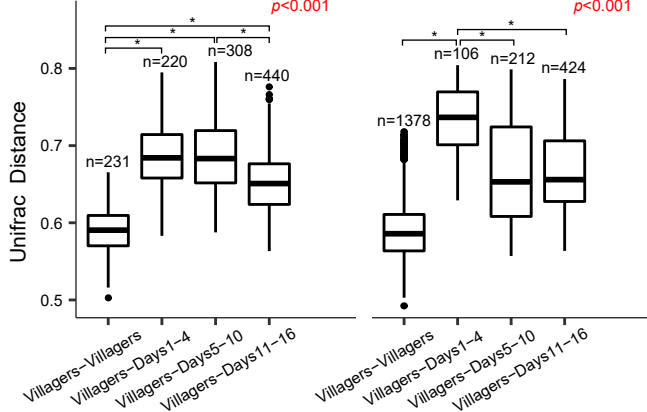

Supplement: FIG S3 [file sph004182633sf3.pdf]

### A. Fecal

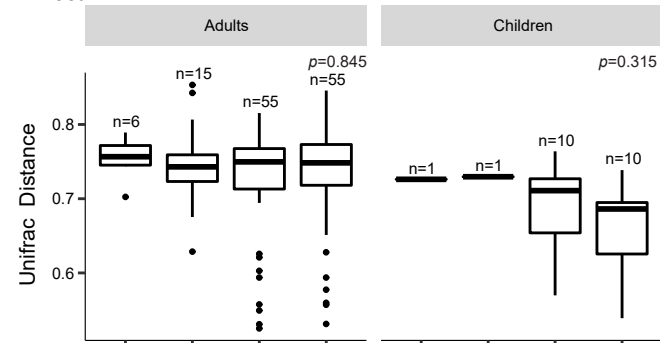

### B. Nasal

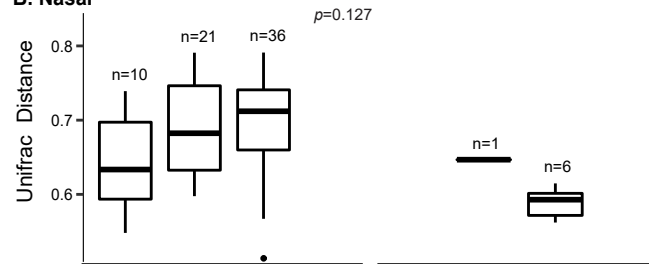

### C. Oral

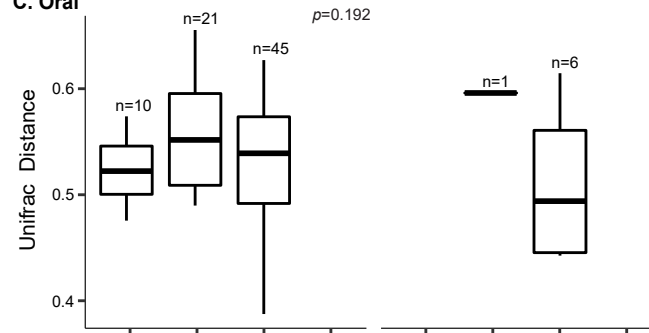

### D. Skin

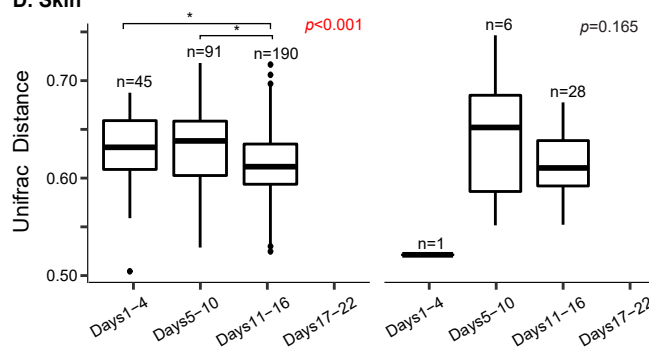

Supplement: FIG S4 [file sph004182633sf4.pdf]

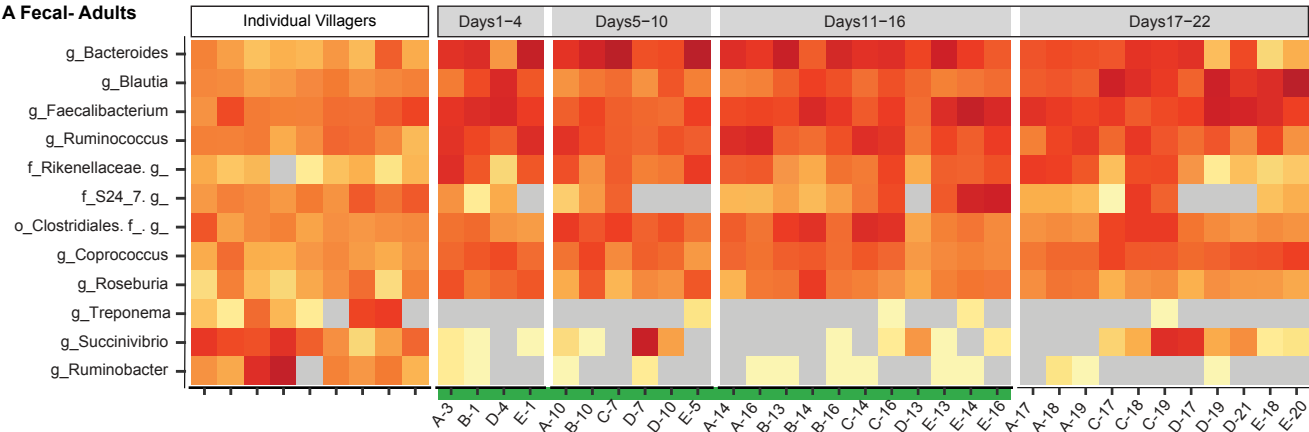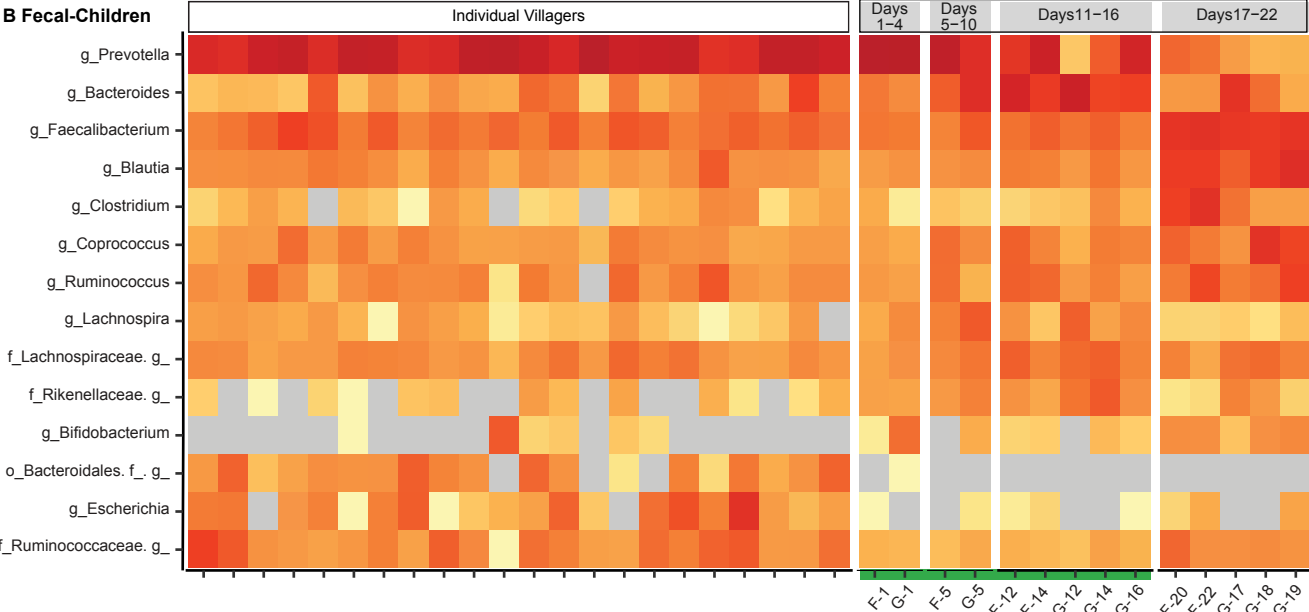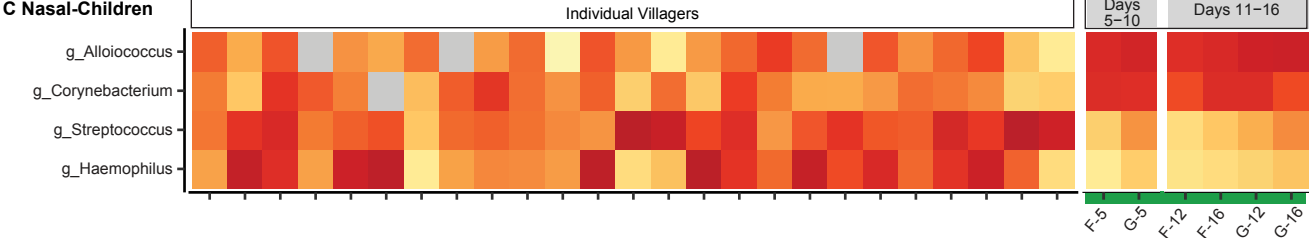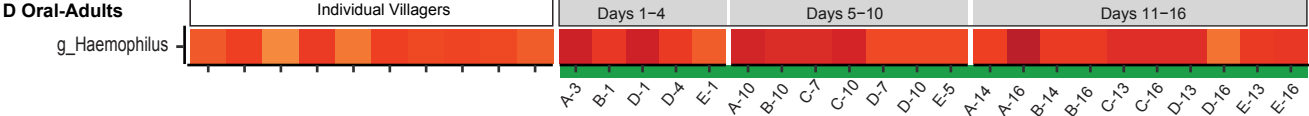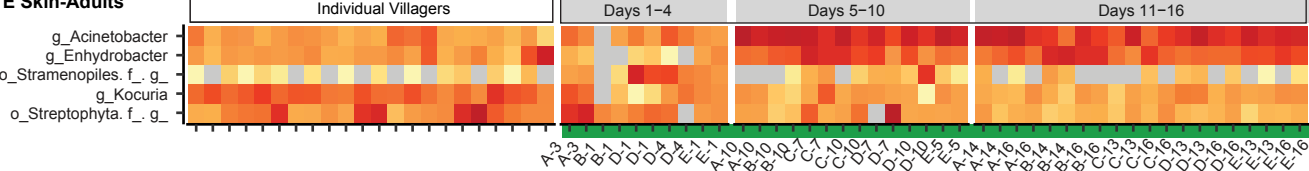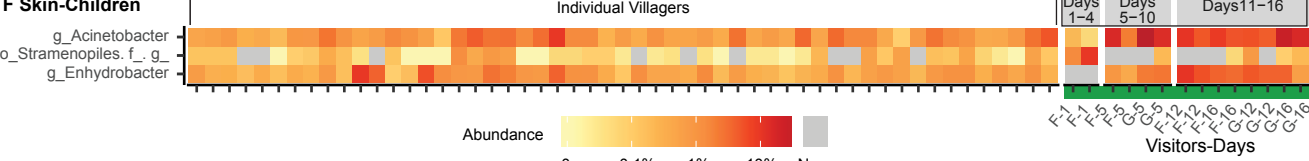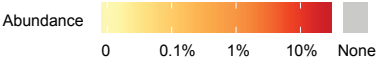

Visitors-Days

Supplement: FIG S5 [file sph004182633sf5.pdf]

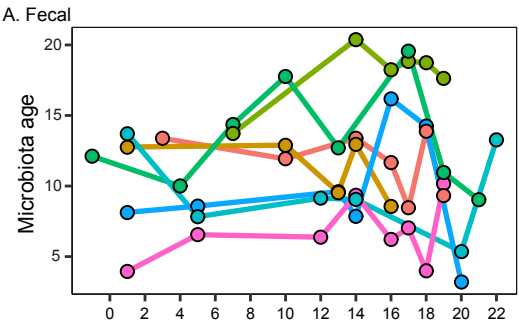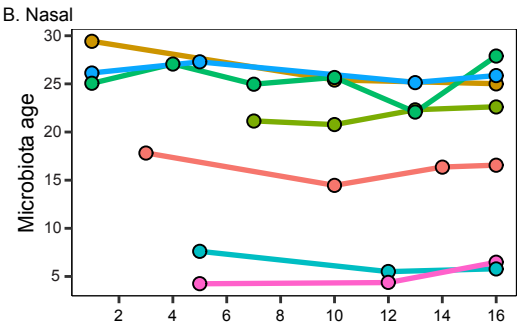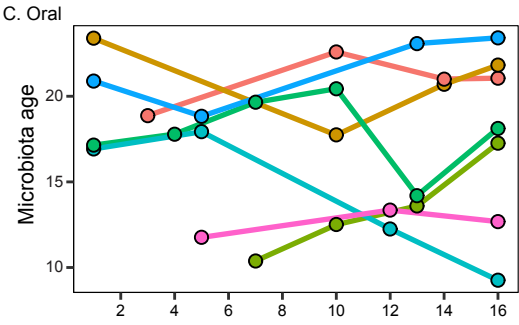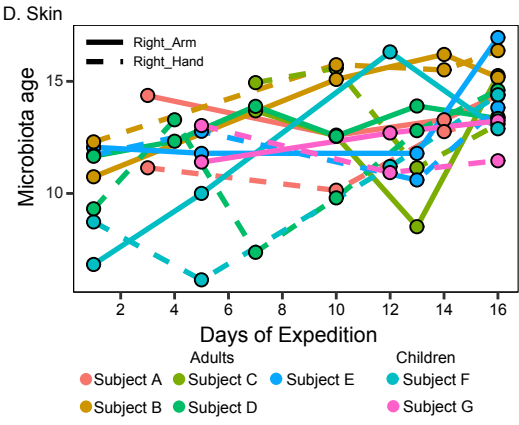

Supplement: FIG S6 [file sph004182633sf6.pdf]

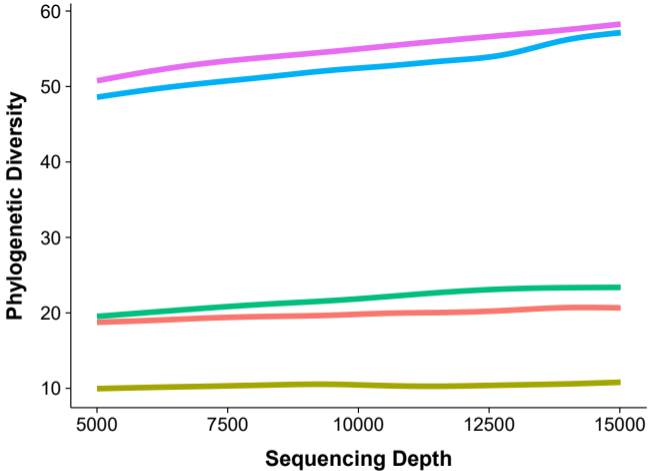

Feces Mouth Nose Right\_Arm Right\_Hand

Supplement: FIG S8 [file sph004182633sf8.pdf]
