# Supplementary material for: Changes in the Gut Microbiota of Urban Subjects during an Immersion in the Traditional Diet and Lifestyle of a Rainforest Village
Source: mSphere. 2018 Aug 29;3(4):e00193-18. doi: 10.1128/mSphere.00193-18 (PMC6115531; doi:10.1128/mSphere.00193-18)
Supplement: TABLE S2 [file sph004182633st2.docx]

Table S2 Microscopy detection of parasites in fecal samples from villagers (4 out of 11 adults and 10 out of 27 children).

| **Age Group** | **Adults** | | | | **Children** | | | | | | | | | |
| --- | --- | --- | --- | --- | --- | --- | --- | --- | --- | --- | --- | --- | --- | --- |
| **Family_ID** | **16** | **16** | **17** | **21** | **3** | **5** | **10** | **12** | **12** | **17** | **17** | **18** | **21** | **25** |
| **Subject_ID** | **79** | **80** | **81** | **99** | **17** | **39** | **52** | **65** | **67** | **84** | **85** | **88** | **112** | **127** |
| Ancylostomide_Eggs | No | No | No | No | No | Yes | Yes | No | No | Yes | No | No | No | No |
| Ascaris_lumbricoides_Eggs | No | No | No | No | Yes | No | Yes | No | Yes | No | No | No | No | No |
| Giardia_lamblia_Cysts | No | Yes | Yes | No | No | Yes | No | Yes | No | No | No | No | Yes | Yes |
| Blastocystis_spp | No | Yes | Yes | Yes | No | Yes | Yes | No | No | No | No | No | No | No |
| Iodamoeba_butschlii_Cysts | No | No | No | No | No | No | No | No | No | Yes | No | No | No | No |
| Endolimax_nana_Cysts | No | Yes | Yes | No | Yes | No | No | Yes | Yes | Yes | No | No | No | No |
| Entamoeba_histolytica_dispar | No | Yes | No | Yes | No | No | No | Yes | Yes | Yes | No | No | No | No |
| Entamoeba_coli_Cysts | Yes | Yes | No | No | No | No | No | No | No | No | No | No | No | No |
| Hymenolepis_nana_Eggs | No | No | No | No | No | No | No | No | No | No | Yes | No | No | No |
| **Number of parasite detected** | 1 | 5 | 3 | 2 | 2 | 3 | 3 | 3 | 3 | 4 | 1 | 0 | 1 | 1 |
