# Supplementary material for: Changes in the Gut Microbiota of Urban Subjects during an Immersion in the Traditional Diet and Lifestyle of a Rainforest Village
Source: mSphere. 2018 Aug 29;3(4):e00193-18. doi: 10.1128/mSphere.00193-18 (PMC6115531; doi:10.1128/mSphere.00193-18)
Supplement: FIG S7 [file sph004182633sf7.pdf]

2015 wet season

September					October																					
					Days 1-4				Days 5-10						Days 11-16						Days 17-22					
26	27	28	29	30	1	2	3	4	5	6	7	8	9	10	11	12	13	14	15	16	17	18	19	20	21	22
Caracas					Kanarakuni village																Caracas					
